# Operations on the Nasal Septum

**Published:** 1909-02-20

**Authors:** 


### Laryngology and Rhinology.

### OPERATIONS ON THE NASAL SEPTUM.

Apart from such operations as the removal of malignant tumours and the pulling out of polypi with forceps, the surgery of the nose is of recent growth, and has only within the last few years attained any considerable degree of refinement. This is markedly true of operations undertaken for the relief of nasal obstruction. Until some ten or fifteen years ago it was a common practice to remove the inferior turbinal body from the narrow nostril to relieve obstruction due to a deflection of the septum, and to pull out roughly the whole of these unfortunate structures with the spokeshave or even with forceps in such cases. However, it gradually became more clearly understood that the vascular and glandular structure of the turbinals are of the greatest importance in warming, moistening, and filtering the inspired air, and that their too free removal is followed by the unpleasant consequences of dryness of the throat and crusting within the nose. It was found that the best results in the treatment of nasal obstruction are to be obtained by operating on the septum when the obstruction was due to deformity of this structure. But deflections of the septum are no easy matter to deal with. In severe cases a hole was sometimes punched through it, and one occasionally meets patients in whom this operation has been performed; but the results were not good, for all the air had then to pass through one choana, and formation of crusts on the margin of the perforation often caused discomfort.

Attempts to break the septum and to make it heal in corrected position by splinting usually ended in failure, for the septum is too resilient to fracture properly and readily returns to its old deformity on removal of the splint. It was found necessary to divide the septum with cutting-forceps, and several fairly successful operations on this plan have been devised. Asch's operation consists in making a crucial incision with two special cutting-forceps, breaking the bases of the triangular flaps thus formed, and then inserting a splint on the convex side; it is useful in cases of simple cup-shaped deflection, but badly adapted for elongated deflections or irregular deformities. Moure's operation is much more generally useful, and was the method chiefly in favour before the introduction of the submucous resection. One incision is made from before backwards close to the floor of the nose, and the other high up above the deflection; the posterior part of the septum is then broken with flat forceps, and the splint inserted and kept in position for ten to fourteen days.

Spurs were removed with the saw, and the first advance towards the submucous resection was begun when an attempt was made to preserve the muco-periosteum over the spur. The Kreig-Bonninghaus operation consisted in cutting away the entire deflected portion of the septum together with the mucous lining of one side; the results were good, but dryness and crusting were troublesome until the lengthy process of healing was complete. Submucous resection was evolved when the muco-periosteum of both sides was left, the deflected portions only of the cartilage and bone being removed. This operation, though not technically easy, gives most excellent results, and has practically superseded all other operations on the nasal septum. It is adapted to every form of deformity, and has the great advantage of leaving the septum thinner than before. In most cases of deflection the septum is not only bent, but also thickened, and often with a ridge or spur on both sides; but by this method the thickening, as well as the deflection, is removed. Again, it is now generally recognised that spurs are not simple excrescences, but are rather the thickened apex of a deflection, and that the best results are therefore obtained by removing them submucously as part of the general deformity. Healing, also, is very rapid, practically no after-treatment is required, and the patient suffers the minimum of discomfort after the operation.

Briefly, the procedure consists in removing as much of the septal cartilage and bone as is deformed from between its two layers of muco-periosteum. An incision, which may be straight, curved, or L-shaped, is made down to the cartilage at the anterior part of the deflection; the position of this can be modified according to the shape of the deformity, but it is usually more or less vertical and made on the convex side of the deviation. In complicated cases a further incision may sometimes be made with advantage along the summit of a prominent ridge, and occasionally an incision may be made at different spots on the two sides, for it is better to cut through the mucosa than to tear it accidentally. The muco-periosteum is then carefully raised over the whole extent of the deflection; it is very important to attempt the separation right on the cartilage beneath the periosteum, for the other layers are much more firmly adherent. The cartilage is then carefully divided in front without injury to the opposite mucosa, and the separation continued on the other side, the whole proceeding being carried out through the one nostril. A long speculum is then introduced to keep the soft parts out of the way, and the cartilage and bone are removed. The former is best excised with Ballenger's swivel-knife, and the latter largely with punch-forceps. The maxillary crest, which is hard and frequently thickened, usually needs a small chisel. The naris, usually only of the convex side, is then lightly plugged for 24 hours to prevent accumulation of blood between the two layers, and treatment ends with its removal.

The chief practical point of the technique is to control completely all bleeding, for a very little obscures the view. Under chloroform anæsthesia this is apt to be troublesome; adrenalin must be thoroughly applied for 20 or 30 minutes previously, and it is well to inject a little beneath the muco-periosteum just before beginning. The patient should be almost in the sitting position. The operation can often be most conveniently done under local anæsthesia, injecting adrenalin with cocaine or one of its substitutes, of which novocaine may be recommended, after a surface application of cocaine.

